# Determination of MIC Distribution and Mechanisms of Decreased Susceptibility to Bedaquiline among Clinical Isolates of Mycobacterium abscessus

**DOI:** 10.1128/AAC.00175-18

**Published:** 2018-06-26

**Authors:** Bing Li, Meiping Ye, Qi Guo, Zhemin Zhang, Shiyi Yang, Wei Ma, Fangyou Yu, Haiqing Chu

**Affiliations:** a Department of Respiratory Medicine, Shanghai Pulmonary Hospital, Tongji University School of Medicine, Shanghai, China; b Tongji University School of Medicine, Shanghai, China; c State Key Laboratory of Microbial Metabolism, Shanghai Jiao Tong University, Shanghai, China; d School of Life Sciences & Biotechnology, Shanghai Jiao Tong University, Shanghai, China; e Department of Clinical Laboratory Medicine, Shanghai Pulmonary Hospital, Tongji University School of Medicine, Shanghai, China; f Shanghai Key Laboratory of Tuberculosis, Shanghai Pulmonary Hospital, Tongji University School of Medicine, Shanghai, China

**Keywords:** Mycobacterium abscessus, decreased susceptibility, antibiotic resistance, bedaquiline, susceptibility testing

## Abstract

Chemotherapeutic options against Mycobacterium abscessus infections are very limited. Bedaquiline, a new antituberculosis (anti-TB) drug, is effective for the treatment of multidrug-resistant TB. However, few data are available on bedaquiline for treatment of M. abscessus infections. In this study, we determined the profile for *in vitro* susceptibility of M. abscessus clinical isolates to bedaquiline and investigated the potential molecular mechanisms of decreased susceptibility. A total of 197 M. abscessus clinical isolates were collected from sputum and bronchoalveolar fluid of patients with lung infections. Standard broth microdilution test revealed that bedaquiline exhibited high *in vitro* killing activity against M. abscessus isolates, with a MIC_50_ of 0.062 and a MIC_90_ of 0.125 mg/liter. Whole-genome sequencing data showed that no nonsynonymous mutation occurred in *atpE*, the gene encoding the bedaquiline-targeted protein. However, of 6 strains with decreased susceptibility of bedaquiline (MIC = 0.5 to 1 mg/liter), 3 strains had nonsynonymous mutations in *mab_4384*, the gene encoding the repressor of efflux pump MmpS5/MmpL5. Quantitative reverse transcription-PCR (qRT-PCR) analysis showed that the expression of MmpS5/MmpL5 in the group with decreased susceptibility to bedaquiline was significantly higher than in those with medium MICs (MIC = 0.125 to 0.5 mg/liter) or in the low-MIC group (MIC ≤ 0.062 mg/liter). Two isolates with increased MICs did not show overexpression of MmpS5/MmpL5, which could not be explained by known molecular mechanisms. This is the first report showing the association of MmpS5/MmpL5 with decreased bedaquiline susceptibility in M. abscessus clinical isolates and suggesting the presence of other, yet-to-be identified mechanisms for decreased bedaquiline susceptibility in M. abscessus.

## INTRODUCTION

Infections caused by nontuberculous mycobacteria (NTM) have been increasing dramatically around the world in recent years (1). Mycobacterium abscessus is one of the most commonly detected pathogens among rapidly growing NTM, and it often causes high morbidity and mortality among patients with chronic lung diseases such as bronchiectasis, chronic obstructive pulmonary disease (COPD), and cystic fibrosis (CF) (2, 3). Human-to-human transmission of M. abscessus infection was reported recently, making the problem more disconcerting (1, 4). Because M. abscessus is intrinsically resistant to various kinds of antimicrobials available in clinical practice, the treatment options for M. abscessus infections are limited (5). The 2007 American Thoracic Society Guideline recommended a long period (at least 1 year) of a combination treatment regimen including macrocyclic lactones (clarithromycin or azithromycin), aminoglycosides (amikacin), and β-lactams (cefoxitin or imipenem) for M. abscessus infections (3). However, a meta-analysis in 2017 showed that the curative effect of this regimen is still very limited, with effective rates of 34% to 54% for newly diagnosed M. abscessus pulmonary disease, and 20% for refractory disease (6). Thus, development of new drugs for the treatment of M. abscessus infections is an urgent need.

Bedaquiline, a new diarylquinoline antituberculosis (anti-TB) drug, targets the c subunit of ATP synthase and exerts an antibacterial effect by blocking ATP synthesis (7–9). Bedaquiline is effective for the treatment of Mycobacterium tuberculosis organisms with very low MICs. It was approved by the Food and Drug Administration and the European Medicines Agency for the treatment of multidrug-resistant tuberculosis (MDR-TB) in December 2012 (10).

One clinical report demonstrated that bedaquiline also possesses potential therapeutic activity in patients with severe M. abscessus lung disease, indicating that bedaquiline could be considered as a salvage therapy for M. abscessus infections (11). However, the MIC data for bedaquiline against M. abscessus are limited, and no bedaquiline susceptibility breakpoint is available for M. abscessus so far. The mechanism of bedaquiline nonsusceptibility is virtually unknown (12, 13). In this study, we determined the *in vitro* profile of susceptibility of M. abscessus clinical isolates to bedaquiline and investigated the potential molecular mechanisms underlying the decreased susceptibility.

## RESULTS

### Bedaquiline susceptibility profile of M. abscessus clinical isolates.

**FIG 1:**
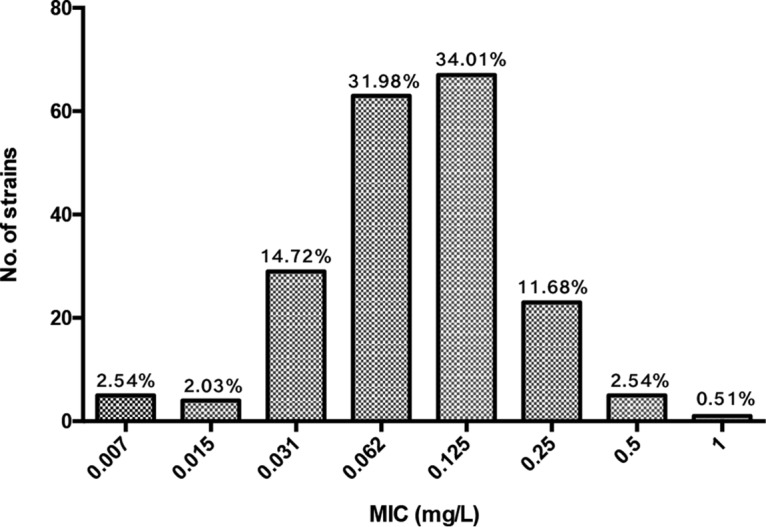
*In vitro* bedaquiline susceptibility profile of M. abscessus clinical isolates. *Mycobacterium peregrinum* ATCC 700686 and Staphylococcus aureus ATCC 29213 served as control reference strains.

A total of 197 M. abscessus strains were isolated from sputum and bronchoalveolar lavage fluid samples during the period from January 2012 to December 2016. Of these, 163 strains were Mycobacterium abscessus subsp. *abscessus* and 34 strains were Mycobacterium abscessus subsp. *massiliense* (Table S1). The MICs of bedaquiline against M. abscessus clinical isolates ranged from 0.007 to 1 mg/liter, with a MIC_50_ and MIC_90_ of 0.062 and 0.125 mg/liter, respectively (Fig. 1). This result suggested that bedaquiline exhibited a high *in vitro* killing activity against M. abscessus isolates.

### Sequence analysis of *atpE* and *mab_4384*.

Strains were divided into three groups according to the levels of bedaquiline susceptibility: those showing low MICs (≤0.062 mg/liter [*n* = 101]), medium MICs (0.125 to 0.25 mg/liter [*n* = 90]), and high MICs (0.5 to 1 mg/liter [*n* = 6]) (overall MIC and mutation information for all the strains is listed in Table S1). Among the 197 strains used in this study, no nonsynonymous mutation was found in *atpE*, the gene encoding the bedaquiline-targeted protein, suggesting that the decrease in bedaquiline susceptibility of these clinical isolates was not due to the *atpE* gene. This notion is consistent with previous reports (9, 14, 15).

It was reported that in M. tuberculosis, the MmpS5/MmpL5 efflux pump is involved in bedaquiline resistance. Mutations in the gene for the repressor of *mmpS5/mmpL5*, *rv0678*, lead to overexpression of *mmpS5/mmpL5* and subsequently contribute to bedaquiline resistance in these M. tuberculosis strains (16). Mab_4383/Mab_4382 and Mab_4384 in M. abscessus are homologous to MmpS5/MmpL5 and Rv0678 in M. tuberculosis. Sequence comparative analysis of Mab_4384 among the 197 strains was performed. Strains with decreased susceptibility (MICs of 0.5 to 1 mg/liter) possessed mutations of A169S, Q215R, H7R, and E142K (Table 1). A169S, H7R, and E142K are located in the functional domain of Mab_4384, which may affect the function of Mab_4384 and subsequently impact the expression of efflux pump gene *mmpS5/mmpL5*. In contrast, Q215R is located outside the functional domain of Mab_4384. Q215R was also present in strains with low MICs (≤0.062 mg/liter), indicating that this mutation did not affect the function of Mab_4384. More interestingly, more than 50% of strains with low and medium MICs harbored a deletion of *mab_4384*, but none of the strains with high MICs did (Table 1). Further sequence analysis revealed that *mmpS5/mmpL5* was absent in all strains with the *mab_4384* deletion (data not shown). This result suggested that the deletion of *mab_4384*, and efflux pump gene *mmpS5/mmpL5*, may contribute to the susceptibility of M. abscessus to bedaquiline.

**TABLE 1:** Mutation information for Mab_4384 among 197 M. abscessus strains used in this study

Mutation(s) of Mab_4384	No. (%) of isolates with mutation in group with the indicated MIC (mg/liter)		
	0.5–1 (*n* = 6)	0.125–0.25 (*n* = 90)	≤0.062 (*n* = 101)
N1T	0 (0)	1 (1.1)	2 (2.0)
G125D, Q215R	0 (0)	2 (2.2)	1 (1.0)
A152E	0 (0)	1 (1.1)	1 (1.0)
A169S	1 (16.7)	0 (0)	0 (0)
Q215R	1 (16.7)	4 (4.4)	3 (3.0)
V31I	0 (0)	3 (3.3)	1 (1.0)
V31I, D120N	0 (0)	1 (1.1)	0 (0)
V5 M, H7R, E142K, A217S	0 (0)	1 (1.1)	0 (0)
H7R, E142K	1 (16.7)	5 (5.6)	0 (0)
W88G	0 (0)	1 (1.1)	2 (2.0)
Deletion	0 (0)	47 (52.2)	62 (61.4)
No mutation	3 (50.0)	24 (26.7)	29 (28.7)

### Transcriptional analysis of efflux pump MpS5/MmpL5.

**FIG 2:**
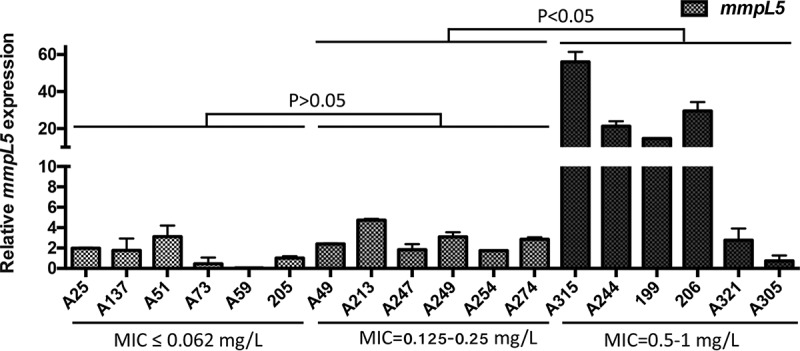
Quantitative reverse transcription-PCR (qRT-PCR) assessment of transcriptional level of *mmpL5*. Error bars represent the standard errors of the data points. *t* test was used to test the difference among groups.

We hypothesize that mutations of Mab_4384 in the isolates with high MICs lead to increased expression levels of the efflux pump gene *mmpS5/mmpL5* and contribute to decreased bedaquiline susceptibility. Isolates with bedaquiline MICs of 0.5 to 1 (*n* = 6), and 6 randomly selected isolates from the low- and medium-MIC groups, were subjected to quantitative reverse transcription-PCR (qRT-PCR) analysis for *mmpS5/mmpL5* expression. As shown in Fig. 2, the expression levels of *mmpS5/mmpL5* in the high-MIC group were significantly higher than those in the medium-MIC and low-MIC groups. Two isolates, A321 and A305, with MICs of 0.5 to 1 mg/liter did not show overexpression of MmpS5/MmpL5. These two isolates also did not have nonsynonymous mutations in *atpE*. Thus, other, yet-to-be identified mechanisms are likely present in these two isolates that contribute to the decreased bedaquiline susceptibility.

## DISCUSSION

Chemotherapeutic therapies against infections caused by M. abscessus are often unsuccessful due to its intrinsic resistance to most antibiotics. New drugs, especially new anti-TB drugs, against M. abscessus infections have brought new hope for treating M. abscessus infections. With the advantages of oral delivery, bedaquiline has been considered as a prospective drug in the treatment of M. abscessus infections (17). Thus, clinical data for *in vitro* susceptibility of M. abscessus to bedaquiline are urgently needed.

In this study, we collected 197 M. abscessus clinical isolates in Shanghai, China. We found that bedaquiline exhibited high *in vitro* killing activity against M. abscessus, with a MIC_50_ of 0.062 and MIC_90_ of 0.125 mg/liter. In contrast to our data, Pang et al. reported that bedaquiline has a moderate antibacterial activity against M. abscessus, with a MIC_50_ of 0.13 and MIC_90_ of >16 mg/liter (9). The difference may be due to the potential exposure of M. abscessus to second-line anti-TB drugs in the study by Pang et al., such as clofazimine, which gains cross-resistance with bedaquiline. In this study, we tested the MIC of clofazimine to M. abscessus and found that it was below 1 mg/liter, supporting the absence of bedaquiline exposure.

Based on our data and those of others (14, 15, 17), bedaquiline showed a high antibacterial activity at a very low concentration (<0.1 mg/liter). In addition, bedaquiline can maintain a mean plasma concentration of 0.6 mg/liter at standard oral doses (18), and it can be extensively distributed to tissues, including the lungs, according to the pharmacokinetic studies (19). In one M. abscessus-infected mouse model, bedaquiline significantly reduced the bacterial burden in the lungs after 4 days of treatment (20). When bedaquiline was used as salvage therapy for M. abscessus infection, there was clinical improvement in the early stage of treatment, with a sustained reduction of bacterial load in sputum and no severe side effects (11). Therefore, bedaquiline could be an effective alternative in the multidrug therapy of M. abscessus infections. However, some negative results also merit attention. Lerat et al. reported that bedaquiline showed almost no activity in nude mice (21), and in the previously mentioned salvage therapy trial, long-term bedaquiline treatment efficacy was shown to not be ideal (11). Furthermore, according to Alexander and coworkers, even very low bedaquiline MICs that might ostensibly be viewed as indicating susceptibility may be associated with treatment failure (22). More bactericidal activity trials are needed to confirm the usefulness of bedaquiline in M. abscessus infections treatment.

The emergence of bedaquiline resistance and treatment failure in TB highlights the importance of rational use of bedaquiline in clinical practice as well as monitoring bedaquiline susceptibility of the pathogen during the course of therapy. Understanding of the mechanisms of bedaquiline resistance is necessary to direct clinical therapeutic choices and reduce the occurrence of resistance (12). Currently known mechanisms of bedaquiline resistance are as follows. (i) Mutations within the target gene *atpE*, including those yielding A28V, A63P, I66M, A28P, G61A, D28N, and A63V changes, prevent bedaquiline from binding to the c subunit of AtpE and finally exert an antibacterial effect by blocking ATP synthesis. These target-based mutations can increase bedaquiline MICs 8- to 133-fold against M. tuberculosis after *in vitro* exposure to bedaquiline (19, 23, 24). (ii) Mutations in *Rv0678*, a transcriptional repressor of efflux pump MmpS5/MmpL5, cause 2- to 8-fold increases of bedaquiline MICs in M. tuberculosis isolates after both *in vitro* and *in vivo* exposure to bedaquiline (25–28). (iii) Mutations in *pepQ* were also reported conferring a 4-fold increase of bedaquiline MIC against M. tuberculosis, though the gene function was unclear (29). (iv) During the bedaquiline treatment course, *mmpT5* mutations in Mycobacterium intracellulare were found to be associated with 2- to 8-fold bedaquiline MIC increasse (22). However, no homologs of PepQ and MmpT5 were found in 197 genomes in this study.

Little is known about mechanisms of bedaquiline resistance in M. abscessus. A report in 2017 by Dupont and colleagues showed construction of an *atpE* mutant of bedaquiline-sensitive M. abscessus and demonstrated that mutation in *atpE* can lead to bedaquiline resistance (15). Pang and colleagues identified 66 bedaquiline-resistant strains from 381 M. abscessus clinical isolates, of which 15 had *atp*E mutations. However, all of the mutations were synonymous (9). No nonsynonymous *atpE* mutation has been found among clinical isolates of M. abscessus. This remains true in our study: no *atpE* mutation was found in all the 197 clinical M. abscessus isolates. This is different from the mechanisms of bedaquiline resistance in M. tuberculosis (23).

Overexpression of MmpS5/MmpL5 caused by *Rv0678* mutation was prevalent in MDR M. tuberculosis isolates from patients treated with bedaquiline or without documented prior use of clofazimine or bedaquiline (28), indicating that elevated expression of MmpS5/MmpL5 contributed to both intrinsic and acquired bedaquiline resistance in M. tuberculosis. Currently, no information is available about MmpS5/MmpL5 expression in bedaquiline-nonsusceptible M. abscessus clinical isolates. Our study is the first showing a role for MmpS5/MmpL5 in decreased bedaquiline susceptibility in M. abscessus clinical isolates (4/6 [66.7%]). Furthermore, we showed that the decreased bedaquiline susceptibility is the result of mutation in the repressor gene *mab_4384*. None of the MmpS5/MmpL5-overexpressing M. abscessus strains had been exposed to bedaquiline or clofazimine before. Therefore, overexpression of MmpS5/MmpL5 appears to be associated with intrinsic bedaquiline resistance in M. abscessus clinical isolates. There was one isolate, A315, with a bedaquiline MIC of 1 mg/liter that showed an extremely high level of *mmpS5/mmpL5* expression. Sequence comparative analysis of this clone showed no mutation in *mab_4384*, indicating the presence of other unknown regulator for *mmpS5/mmpL5* that remains to be investigated. In addition, we showed 2 isolates with elevated bedaquiline MICs (A321 and A305) without overexpression of MmpS5/MmpL5 or *atpE* mutation, suggesting the presence of an MmpS5/MmpL5-independent pathway which could not be explained by current known mechanisms. We are currently in the process of investigating the remaining molecular mechanisms in these strains.

## MATERIALS AND METHODS

### Isolation of M. abscessus clinical strains.

A total of 197 M. abscessus isolates were collected from sputum and bronchoalveolar lavage fluid samples of patients with lung infections in Shanghai Pulmonary Hospital from January 2012 to December 2016. Isolates were preliminarily screened for NTM by both MGIT960 medium culture and *p*-nitrobenzoic acid test, followed by molecular identification of M. abscessus by sequencing of the *rpoB* and *erm*(41) genes (5, 30). All isolates were then stored at −80°C until use.

### Bedaquiline susceptibility test.

Bedaquiline (Biopharmaleader, China) susceptibility was determined by the broth microdilution method according to CLSI document M24-A2 (31). *Mycobacterium peregrinum* (ATCC 700686; American Type Culture Collection, Manassas, VA) and Staphylococcus aureus (ATCC 29213; American Type Culture Collection) served as the control reference strains.

### Whole-genome sequencing and comparison of *atpE* and *mab_4384*.

In this study, 35 strains isolated in 2016 were sequenced. DNA extraction, library construction, and sequencing were performed as we described previously (32). The whole genomes of the other 162 strains isolated during 2012 to 2015 were published by us previously (32). Sequences of *atpE* (*mab_1448*) and *mab_4384* were extracted from the sequencing data. Sequences were aligned to the homologous sequences of the reference mycobacterial strain ATCC 19977 by BLAST (33).

### RNA extraction and qRT-PCR.

RNA samples were extracted from mid-log-phase bacterial cultures according to the protocols recommended by Medjahed and Singh (34). cDNA was synthesized using the RT reagent kit with gDNA Eraser (TaKaRa, Shiga, Japan). Quantitative reverse transcription-PCR (qRT-PCR) was performed using SYBR Premix ExTaq (TaKaRa) on a 7500 real-time PCR system (Applied Biosystems, Carlsbad, CA). Reactions were repeated in triplicate and the fold change in gene expression was calculated as previously described (35). Clinical M. abscessus strain 205, with a bedaquiline MIC of 0.007 mg/liter, was used as the reference strain for the gene expression analysis. PCR primer pairs for amplification of *mmpL5* and the endogenous reference gene *sigA* were *mmpL5*_RT_F (AGAGCAGCGACGGAAAGG)/*mmpL5*_RT_R (TTGGTCTGCCGAGGTTGTC) and *sigA*_RT_F (AGCGTGAGCTGCTACAGGAC)/sigA_RT_R (TGGATTTCCAGCACCTTCTC).

### Accession number(s).

The accession numbers for the 35 M. abscessus isolates sequenced in this study are available at DDBJ/ENA/GenBank under BioProject no. PRJNA448987.

## Supplementary Material

Supplemental material
