# Supplementary material for: Determination of MIC Distribution and Mechanisms of Decreased Susceptibility to Bedaquiline among Clinical Isolates of Mycobacterium abscessus
Source: Antimicrob Agents Chemother. 2018 Jun 26;62(7):e00175-18. doi: 10.1128/AAC.00175-18 (PMC6021634; doi:10.1128/AAC.00175-18)
Supplement: Supplemental material [file supp_62_7_e00175-18__index.html]

Supplemental material 

# Determination of MIC Distribution and Mechanisms of Decreased Susceptibility to Bedaquiline among Clinical Isolates of Mycobacterium abscessus

## Supplemental material

- Supplemental file 1 -

  Table S1

  XLSX, 16K
